# The Phonological Development of Mandarin Voiceless Affricates in Three- to Five-Year-Old Children

**DOI:** 10.3389/fpsyg.2022.809722

**Published:** 2022-03-10

**Authors:** Junzhou Ma, Yezhou Wu, Jiaqiang Zhu, Xiaoxiang Chen

**Affiliations:** ^1^School of Foreign Languages, Taizhou University, Taizhou, China; ^2^School of Foreign Languages, Hunan University, Changsha, China

**Keywords:** voiceless affricates, Mandarin, children, phonological acquisition order, phonological development

## Abstract

This study investigates the phonological development of Mandarin voiceless affricates produced by Mandarin-speaking children. Thirty-six monolingual Mandarin-speaking children and twelve adults participated in a speech production task. Auditory-based transcription analysis and acoustic analysis were utilized to quantify the relative order of affricate acquisition. Both methods yielded earlier acquisition of alveopalatal affricates at age three than retroflex and alveolar affricates, whereas they differed in the acquisition order of retroflex and alveolar affricates. The former revealed that both retroflex and alveolar affricates were acquired at age five, regardless of aspiration, while the latter yielded earlier acquisition of retroflex than alveolar affricates. Possible reasons for the discrepancy are discussed in relation to the different nature of the two methods. Overall, the observed acquisition order of Mandarin voiceless affricates suggests that child speech development is a complex process, and is influenced by various factors including oromotor maturation and language-specific phoneme frequencies in the ambient language.

## Introduction

It was once believed that children acquire language seemingly effortlessly at a very young age and show universal trajectories of phonological development, irrespective of the linguistic environment. Within the first year of life, children experience similar processes, from producing vowel-like cooing to consonant-vowel structure (babbling). Thereafter they continue to produce vowels, stops, and nasals, followed by fricatives and liquids (Jakobson, 1968; Locke, 1983). This developmental trajectory indicates common biological constraints that influence the acquisition process. However, exposure to a particular language could also affect the process of phonological development. As a result, children produce speech sounds at a different speed and varying rate (Vihman, 1993; Li and Munson, 2016), reflecting a combination of factors such as articulatory complexity, the ambient language, and oromotor maturation (Jakobson, 1968; Locke, 1983; Pye et al., 1987; Ingram, 1988a; de Boysson-Bardies et al., 1989; Stokes and Surendran, 2005). However, the relative contribution of these factors differs across languages. Research into these factors could help deepen our understanding of the phonological development in typically developing children of a given language. The current study seeks to document the acquisition of Mandarin voiceless affricates by combining the power of transcription with acoustic analysis.

### Factors Related to the Order of Phonological Acquisition

Regarding the phonological acquisition order, decades of endeavor have been made in order to provide a satisfactory account. Jakobson’s (1968) postulations are the most influential ones that have been extensively discussed and tested in the literature (So and Dodd, 1995; Amayreh and Dyson, 1998). Jakobson described phonological development as the emergence of phonological contrasts based on an implicational hierarchy. One of his most popular claims is that the distribution of phoneme types in world’s languages would predict the order of phonological acquisition no matter what language children are exposed to. Phoneme types frequently observed in world’s languages are acquired earlier than less frequently observed ones. For instance, vowels, stops, and nasals are widely distributed in world’s languages. They are, therefore, acquired much earlier than fricatives, affricates, and liquids. The frequency of phoneme types in world’s languages reflects the complexity of underlying articulatory mechanism. Phonemes with less articulatory complexity are frequently observed in world’s languages and are expected to be acquired earlier than those with more articulatory complexity. In addition, Jakobson also put forward the “fronting universal” to explain the relative sound acquisition sequence in terms of place of articulation, namely, anterior sounds are acquired earlier compared with posterior ones (Locke, 1983).

Jakobson’s universal acquisition order based on the phoneme distribution in world’s languages is challenged by subsequent studies, showing that it fails to account for the order of phoneme acquisition in particular languages. These studies reveal that the acquisition order is affected by the ambient language, especially language-specific phoneme frequencies (Pye et al., 1987; Ingram, 1988a; Stokes and Surendran, 2005; Edwards and Beckman, 2008; Ambridge et al., 2015; Li and Munson, 2016; Tang et al., 2019b). For example, Pye et al. (1987) conducted a longitudinal study to investigate the phonological development of Quiche in children. The findings showed that /t∫/ was acquired much earlier in spite of its rare distribution in world’s languages, and the order of acquisition positively correlated with the frequency of occurrence. They also found language-specific substitution patterns for the same sounds in children acquiring different languages. They claimed that it was the high frequency of /t∫/ in Quiche that led to its early acquisition, suggesting that language-specific phoneme frequencies affect the acquisition order significantly, and high articulation complexity does not necessarily preclude earlier acquisition.

Given that speech articulation is constrained by anatomical and physiological development, it is therefore reasonable to posit that anatomical maturation also contributes to the acquisition of speech sounds. Research has shown that the development of vocal behavior in the first year of life could be largely attributed to the biology-based developmental function modules (DFMs) that characterizes the speech system (Kent, 2021, 2022). The Lingual Complex DFM, one of the DFMs identified in Kent (2021), mainly comprises the tongue and mandible, the former of which is closely related to production of affricates. There are four submodules including tongue body, coronal raising, dorsal raising, and pharyngeal. In addition, the development of motor control progresses from large-muscle (i.e., arms and legs) to small muscle (i.e., hands and toes) use, and from central (i.e., palm) to peripheral (i.e., fingers) body parts (McBryde and Ziviani, 1990; Butterworth et al., 1997; Wallace and Whishaw, 2003), termed *proximal-distal principle*. Based on this principle, Li and Munson (2016) proposed the oromotor maturation hypothesis that sounds involving the tongue body would be acquired earlier than those involving the tongue tip. They further applied this principle to tongue maturation and speculated that children have earlier control over the tongue body than other parts of the tongue (i.e., tongue tip), which accounts for the earlier acquisition of alveopalatal fricatives in diverse languages (Ingram, 1988b; Gibbon, 1999; Li et al., 2011; Li and Munson, 2016), though this sound is infrequently found in world’s languages, in stark contrast to the ideas proposed by Jakobson (1968). Mandarin affricates are characterized with a two-way aspiration contrast across three places of articulation, distinguishing itself from many other languages (Lin and Wang, 1992). The primary difference between alveopalatal affricates and other affricates lies in the involvement of central and large muscles that control the tongue dorsum in comparison with the utilization of the peripheral and relatively smaller muscles responsible for the raising and lowering of the tongue tip. Therefore, this oromotor maturation hypothesis may readily explain the earlier acquisition of alveopalatal affricates.

### Methodological Issues Stemming From Previous Research

Although all these studies have deepened our understanding of various factors that influence phonological development, there are still unsolved issues plaguing research in the field, which might result from the methodological issues in the preceding literature. Therefore, a reliable description of child speech patterns plays a key role in settling the controversies regarding phonological development. Most prior studies on phonological development were based on the transcription method; however, increasing studies have repeatedly pointed out the shortcomings of transcription analysis (e.g., Edwards and Beckman, 2008; Li, 2008; Munson et al., 2010, 2012; Yang, 2014; Li and Munson, 2016; Tang et al., 2019a). For example, listeners perceive speech sounds categorically, ignoring the acoustic differences within the category (Liberman et al., 1957). Children might articulate two different speech sounds which, however, were perceived as a single adult category, because the differences were below adults’ perceptual thresholds and such distinctions were often overlooked by adult transcribers. This phenomenon was termed *covert contrast*, which has been supported by a growing number of studies utilizing acoustic analysis (Scobbie et al., 2000; Li et al., 2009). Due to the coarseness and the inadequate descriptive power of the transcription analysis, it was highly likely to obscure the actual developmental sequence for individuals in childhood. In addition, prior studies primarily targeted the whole phonological system and the sampling of target sounds was not strictly controlled, resulting in unbalanced data. Furthermore, few studies have used statistical analysis to examine phonological development.

### Previous Studies of the Acquisition of Mandarin Voiceless Affricates

Ample studies have been carried out to investigate the order of phonological acquisition in Indo-European languages (e.g., Stoel-Gammon and Cooper, 1984; Pye et al., 1987; Amayreh and Dyson, 1998; Stokes and Surendran, 2005). However, studies of phonological acquisition of Mandarin are much more rare (So and Dodd, 1995; Hua and Dodd, 2000; Si, 2006; Xu et al., 2010). For example, Hua and Dodd (2000) examined the phonological acquisition of Mandarin in general by 129 Mandarin speakers aged 1;6–4;6. The results showed that alveopalatal affricates such as /tɕ/ and /tɕ^h^/ were acquired at age 2;6 (75% criterion), earlier than other affricates, which had not been acquired by the age of 4;6. They concluded that such acquisition order could be accounted for by the phonological saliency of the phonemes in a specific language system, rather than the universal developmental path. Apart from these cross-sectional studies, longitudinal studies were also conducted to explore the phonological development of Mandarin (Si, 2006), revealing that the observed phonological acquisition order diverged from the universal developmental path. For example, the rarely observed alveopalatal affricates were found to be acquired earlier than some other affricates. More recently, Lou (2020) conducted a longitudinal study to examine the phonological development in Mandarin-learning infants, from babbling to the early word period, showing that unaspirated affricates appeared in the early stage of language learning and their use of affricates was higher compared with other 7 European language groups in the literature. The author attributed the early emergence to the high frequency of affricates in their language input, further confirming the influence of the ambient language on phonological acquisition.

Compared with the richness of transcription studies, to the best of our knowledge, few studies adopted the fine-grained acoustic approach to examine phonological development of Mandarin voiceless affricates. Therefore, an objective and controlled study is warranted to complement the power of the transcription analysis. In addition, the rarity of affricates in world’s languages and its distinctive features in Mandarin provide us with a good opportunity to reexamine phonological development in Mandarin speakers.

### The Present Study

This study attempts to investigate the phonetic developmental patterns of Mandarin voiceless affricates in three- to five-year-old Mandarin-speaking children. Both transcription analysis and acoustic analysis were utilized. Auditory-based transcription is widely used in previous studies (e.g., Stoel-Gammon and Cooper, 1984; Pye et al., 1987; Hua and Dodd, 2000), and influential theories are proposed based on transcription analysis (e.g., Jakobson, 1968; Waterson, 1971; Mowrer, 1980; Locke, 1983; Hua and Dodd, 2000), providing a frame for the interpretation of the acoustic patterns. However, acoustic analysis is not as perfect as we expect. Although the acoustic analysis offers fine-grained information on child speech development, it has its own limitations. The validity of the acoustic analysis depends on the selected parameters that could best reflect the child’s articulation, as well as the auditory parameters that most strongly predict listeners’ perceptual judgments. Furthermore, the results of the acoustic analysis still require human interpretation. More importantly, the two methods are generally adopted to address different questions. For example, the acoustic analysis is used to address the question of whether there is any overall statistical difference between two or multiple groups of speech sounds, while the perceptual transcription is made on individual tokens, answering the question about how many tokens sound target-like or if they fall into the native adult’s perceptual categories. However, the two methods do not conflict with each other, and they are more likely to be complementary to offer us a complete picture of child speech development. As an instance, the auditory analysis is mainly used to categorize children’s productions into native categories, thus it has the advantage to classify or count the error type of children’s productions. It could also serve as a data-cleaning process for later acoustic comparisons. The acoustic analysis, however, could quantitatively reveal acoustic differences, which sometimes can be subtle, between groups or speech categories. Taking these issues into consideration, we decide to combine transcription analysis with acoustic analysis in the hope that both methods could complement each other to solidify the sound acquisition sequence in Mandarin-speaking children. We first analyze the affricate productions by adults so as to quantify the acoustic space of Mandarin affricates and guarantee the reliability of the acoustic parameters that distinguish Mandarin affricates in terms of place of articulation. Correspondingly, acquisition here is defined as when children could make phonetic contrasts in the dimension of selected acoustic parameters. Then the acoustic parameters proven to be effective are used to analyze children’s data. Therefore, two research questions are formulated. First, what are the developmental patterns of Mandarin voiceless affricates? Second, what factors in the current theoretical framework could account for the observed phonological acquisition order of Mandarin voiceless affricates?

### Acoustic Measurement

Spectral moments analysis is widely used to analyze acoustic characteristics of obstruents in terms of place of articulation (Jongman et al., 2000; Nissen and Fox, 2009; Li and Gu, 2016; Li and Munson, 2016; Ran, 2017). In order to analyze the acoustic components, the spectral amplitudes of a series of frequency points are generated from the acoustic signal, and the resultant spectra are regarded as random distribution probabilities from which the four spectral moments are calculated. The first spectral moment, spectral mean, is most relevant to the differentiation of affricates in terms of place of articulation of Mandarin affricates (Lee et al., 2014; Li and Gu, 2016; Ran, 2017). Spectral mean reflects the energy concentration. The frequency value of spectral mean has a negative correlation with the length of the front resonating cavity. If the resonating cavity before the constriction point is longer, then the frequency value of spectral mean will be lower. Conversely, a shorter front resonating cavity will lead to a higher value of spectral mean. Hence, alveolar affricates are supposed to have the highest spectral mean, followed by alveopalatal affricates, and then retroflex.

In addition to the consonant-internal factor, consonant-vowel transitional information also contributes to the differentiation between places of articulation. It was found that the second formant (F2) onset could distinguish Mandarin voiceless affricates across places of articulation (Lee et al., 2014; Li and Gu, 2015). F2 onset, defined as the second formant frequency of the onset of the following vowel, has a negative correlation with the length of the resonating cavity behind the constriction point (Nittrouer et al., 1989), which increases as the constriction point moves from the anterior part of the oral cavity to the posterior part. It is expected that alveopalatal affricates have the highest F2 onset values as a result of the tongue posture in the articulation of the frication part. The raised tongue dorsum reduced the length of the back cavity compared with that of retroflex and alveolar affricates, and the size of the back cavity of retroflex is slightly shorter than that of alveolar affricates. Therefore, alveopalatal affricates are expected to show the highest F2 onset values, followed by retroflex, then alveolar affricates.

## Materials and Methods

### Participants

Children aged 3–5 years old and adults as reference participated in the current study. This age range was deliberately selected because all six Mandarin affricates begin to emerge at the age of 3;1–3;6 based on 75% criterion and stabilize after 4;6 (Hua and Dodd, 2000). In addition, our pilot study also revealed that the 2-year-olds could not articulate all affricates, which led to severely unbalanced data. Prior to the experiment, to ensure that all participants were least influenced by dialects and guarantee the homogeneity of speakers’ language background, a trained graduate student of phonetics was asked to evaluate their pronunciation based on a 5-min dialog, in addition to the language background questionnaire. The dialog could also ease the anxiety of the children so that they could participate in the speech production collaboratively. Local residents, speaking Xiang dialect, which has no retroflex in its phonological system, were excluded from this study. Finally, thirty-six qualified children and twelve adults were selected. All participants were monolingual Mandarin-speakers and were equally divided into four groups according to their age range, namely, the three-year-olds (*M*_*age*_ = 3.7 years, *SD* = 0.28), the four-year-olds (*M*_*age*_ = 4.4 years, SD = 0.33), the five-year-olds (*M*_*age*_ = 5.8 years, *SD* = 0.28), and the adult group (*M*_*age*_ = 31.8 years, *SD* = 6.06). Each group had equal number of males and females. All participants were born and raised in China and were monolingual speakers of Mandarin. Their parents and teachers communicated with them in Mandarin in daily life and at school time. Most of them were from middle class family and their parents had a bachelor’s degree, and some of them had a master’s or a doctoral degree. At the time of the experiment, they had visible front incisors and none was reported to have any language, speech, or hearing disorders, confirmed by their parents and teachers. They all passed a pure-tone hearing screening test at 125–8,000 Hz. Both children and adults volunteered to participate in the experiment. All children were given a small gift for their participation. This experiment was approved by the Ethics Committee of Hunan University.

### Stimuli

Eighteen disyllabic words were selected (see Supplementary Appendix A), with one of the six Mandarin voiceless affricates in the word-initial position followed by one of the three corner vowels /a, i, u/. The six voiceless affricates were /ts, ts^h^, tɕ, tɕ^h^, tȿ, tȿ^h^/ as shown in Table 1. In Mandarin, alveolar affricates /ts, ts^h^/ and retroflex /tȿ, tȿ^h^/ cannot occur in front of the high front vowel /i/. Instead, the former two can co-occur with the vowel /ɿ/ and the latter can co-occur with /ʅ/. Given that both /ɿ/ and /ʅ/ are allophones of /i/, /i/ was used to represent these two allophones in the study. Similar to alveopalatal /ɕ/, alveopalatal affricates /tɕ, tɕ^h^/ cannot occur with /u/ due to the phonotactic constraints of Mandarin. Therefore, /y/ was used to substitute for it, and there is a glide between alveopalatal affricates and the nucleus /a/. Despite the fact that Mandarin is a tone language (Zhu et al., 2022), tones were not controlled when selecting the target words due to the issue of familiarity and picturability. The purpose was to the greatest extent to make sure that children in this age range could readily recognize these pictures without heavy cognitive load in case they felt frustrated and withdrew during recording.

**TABLE 1:** Mandarin affricates across place and manner of articulation.

	Alveolar	Alveopalatal	Retroflex
Aspirated	ts^h^	tɕ^h^	tȿ^h^
Unaspirated	ts	tɕ	tȿ

### Procedures

Before the experiment, children’s parents were required to sign a consent form or provide verbal assent/agreement. Demographic and language background information were also provided by the parents. Audio prompts were recorded by a female, who was required to repeat all the target stimuli four times using a child-directed speech. Otherwise, the child participants might be frustrated and unwilling to continue the experiment. The recording was carried out in a quiet room in the day-care center or at their homes. Participants were comfortably seated in front of a laptop (MacBook Air). A supercardioid condenser microphone (Shure SM Beta 87A) connected to a professional solid state compact card recorder (Marantz PMD 660) was fixed on a stand and placed approximately 15 cm from the mouth. Their productions were recorded with a sampling rate of 44.1 kHz and 16-bit quantization. Pictures representing the target words were randomly presented on the computer screen, followed by a pre-recorded audio prompt. All selected 18 pictures were confirmed by parents and children to make sure that they were familiar with them. Otherwise, new pictures would be re-selected. Finally, eighteen pictures were used to represent the target words. The entire experimental stimuli included 72 (18 target stimuli × 4 repetitions) disyllabic words with an affricate in the word-initial position.

Participants were guided to watch the picture and listen to the audio prompt carefully, and they were then required to repeat the audio prompt clearly and naturally without exaggerating or stressing any part of these disyllabic words. Prior to the experiment, they were required to familiarize themselves with the pictures and the experimental procedures. In the case of incorrect or disfluent articulation, the process was interrupted, and they were instructed to repeat the word prompted by the experimenter until a correct one was obtained, and then the experiment proceeded. All participants were allowed to take a break during the recording. Most children chose to take a break and they showed great interests in repeating these words and found the task fun. As for adults, they were told that this experiment was designed for children and they finished the experiment as easily as expected. In general, children finished the task within 15 min and adults within 10 min. Finally, it yielded 3,456 tokens. Those sounds that were unclear, overlapped with the audio prompt, or influenced by background noise were discarded. In total 3,423 sounds were used for the transcription analysis. Furthermore, we excluded 59 mispronounced speech sounds including stops, fricatives, and sounds with incorrect aspiration. Finally, 3,364 sounds were used for statistical analysis.

A trained graduate student of phonetics transcribed all the sounds. Each sound was played *via* Praat (Boersma and Weenink, 2017) such that the transcriber could simultaneously listen to the sound repeatedly while consulting the spectrogram. The transcriber only transcribed the first syllable of the disyllabic words and knew what the target word was. The correctly pronounced sounds were coded as “1” and the incorrectly pronounced sounds were coded as “0.” In addition, the transcriber also recorded the error types of mispronunciations. The transcription was undertaken on a child-by-child basis such that the transcriber had transcribed all the affricate consonants produced by one child before moving to the next one. Then, 20% of the stimuli were randomly selected. Another research assistant transcribed and segmented these selected sounds once again to ensure the inter-reliability of the transcription and measurement. The result of Pearson’s product-moment correlation coefficient analysis showed that the inter-reliability was *r* = 0.9, *p* < 0.001, and *r* = 0.95, *p* < 0.001, respectively, indicating high transcription and segmentation reliability.

Cool Edit Pro 2.1 was used to segment target words from long files and Praat was used to display the waveform and the spectrogram, both of which were simultaneously consulted to segment the words. Since the production of an affricate begins with an alveolar stop /t/, the onset of the affricates was located at the point where stop burst was released. The offset of the frication was labeled at the first upward going zero-crossing that introduced the first periodic wave. The beginning of the following vowel was the same as the offset of the previous obstruent, and the offset of the first syllable was located at the fading of energy or the ceasing of the glottal pulse (Li, 2013; Ma et al., 2018). The maximum view range was 11,025 Hz for both adults and children, and the dynamic range was set at 40 dB. The purpose was to maximize the measurement consistency across tokens. Spectral mean, using spectral moments analysis, was computed from the FFTs. The frication part of the affricates is the same as fricatives. Therefore, spectral moments were calculated at the midpoint of the frication. A 40-ms Hamming window was placed at the middle of the frication of the aspirated affricate, and a 10-ms Hamming window was used for the unaspirated affricate because the midpoint is the most stable portion whereas the onset and offset are often seriously influenced by neighboring sounds. No pre-emphasis was made either. F2 onset was measured at the beginning of the second formant of the vowel. The maximum formant value was set at 5,000 Hz for adult males and 5,500 Hz for adult females. Regarding children’s productions, this value was set at 7,000 Hz. Considering that the second formant of /u/ is relatively much lower, the maximum formant value for measuring /u/ was set at 3,500 Hz (Li, 2008). To avoid miscalculation of F2 onset, visual check of LPC was conducted to confirm the obtained values.

### Statistical Analysis

R (R Core Team, 2018) was used to analyze the obtained data. Linear mixed-effects models were fitted to analyze the differences in transcribed accuracy rates and acoustic parameters. Linear mixed-effects models were fitted *via* lmer functions from LME4 packages (Bates et al., 2015). Barr et al. (2013) recommended that all random slopes should be included in the model to keep it maximal with regard to the random effects structure, and the random-intercept-only linear mixed-effects model could also inflate Type I Error no matter how *p* values were computed (Schielzeth and Forstmeier, 2009). However, it is not realistic to include all random slopes in the model because such model requires a huge data set to estimate variances and covariances correctly with the computing power becoming considerably large (Bates et al., 2015). Subject was included in the model as a random factor and by-subject random slopes for all fixed factors were included to build the initial maximum model. We further compared the maximum model with a simplified one that discarded a factor step by step using ANOVA function in LMERTEST package (Kuznetsova et al., 2017). This ANOVA function from LMERTEST package was also used to compute the *p* values and degree of freedom by adopting Satterthwaite’s approximation. When significant main effects or interaction effects were found, LSMEANS was used to conduct further pairwise comparisons using Tukey adjustment (Lenth, 2016).

## Results

### Adults’ Affricate Production

To assess the effectiveness of the selected acoustic parameters to distinguish three places of articulation, linear mixed-effects models were fitted on the two acoustic parameters for aspirated and unaspirated affricates, respectively. The dependent factors are F2 onset and spectral mean, respectively. The fixed factor is place of articulation (three levels: /ts^h^,ts/, /tɕ^h^,tɕ/, and /tȿ^h^,tȿ/), and by-subject random slopes for the effect of place were included as well. The results are summarized in Supplementary Appendix B.

**FIGURE 1:**
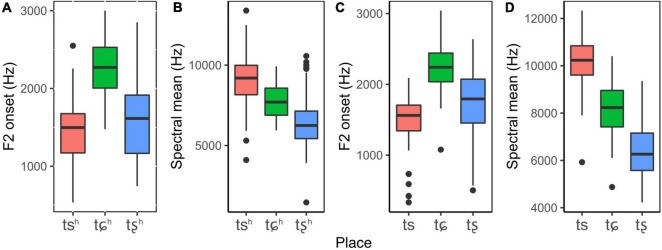
Box plots of F2 onset and spectral mean for aspirated and unaspirated affricates produced by adults. Each box describes the lower quartile, median, and upper quartile.

Figures 1A,B display F2 onset and spectral mean of each aspirated affricate, respectively. F2 onset of /tɕ^h^/ (*M* = 2,259 Hz) is much higher than that of /ts^h^/ (*M* = 1,428 Hz) and /tȿ^h^/ (*M* = 1,557 Hz), and average spectral mean of /ts^h^/ (*M* = 9,097 Hz) is much higher than that of /tɕ^h^/ (*M* = 7,690 Hz) and /tȿ^h^/ (*M* = 6,428 Hz). Results of linear mixed-effects models showed that there were significant main effects of place on F2 onset and spectral mean. *Post hoc* comparisons showed that there were significant differences between places on both F2 onset and spectral mean, except a marginally significant difference between /ts^h^/ and /tȿ^h^/ in terms of F2 onset. These results suggested that F2 onset and spectral mean were robust cues to place of articulation.

Figures 1C,D demonstrate F2 onset and spectral mean of unaspirated affricates. F2 onset of /tɕ/ (*M* = 2,259 Hz) is higher than that of /tȿ/ (*M* = 1,764 Hz) and /ts/ (*M* = 1,506 Hz), and spectral mean of /ts/ (*M* = 10,172 Hz) is much higher than that of /tɕ/ (*M* = 8,195 Hz) and /tȿ/ (*M* = 6,352 Hz). The results of statistical modeling revealed that there were significant main effects of place on F2 onset and spectral mean. *Post hoc* analyses revealed statistically significant differences between three places of articulation, suggesting that F2 onset and spectral mean could successfully distinguish three unaspirated affricates in terms of place of articulation.

### Transcription Analysis

#### Children’s Aspirated Affricates

Table 2 describes the number and percentage of Mandarin voiceless aspirated affricates judged to be correct by the transcriber as a function of age, place, and vowel context. It is found the accuracy rate of aspirated affricates produced by children aged three is much lower than that by children aged four and five.

**TABLE 2:** Number and percentage of aspirated affricates judged to be correct by the transcriber as a function of age, place, and vowel context.

		/ts^h^/		/tɕ^h^/		/tȿ^h^/	
Age	Vowel	Total	Number (percentage) Correct	Total	Number (percentage) Correct	Total	Number (percentage) Correct
Three	/a/	47	29 (62)	48	48 (100)	48	18 (38)
(*n* = 428)	/i/	47	35 (74)	48	44 (92)	48	9 (19)
	/u, y/	48	17 (35)	47	39 (83)	47	33 (70)
	Overall	142	81 (57)	143	131 (92)	143	60 (42)
Four	/a/	48	37 (77)	48	48 (100)	46	31 (67)
(*n* = 424)	/i/	47	44 (94)	46	45 (98)	48	37 (77)
	/u, y/	47	31 (66)	47	44 (94)	47	38 (81)
	Overall	142	112 (78)	141	137 (97)	141	106 (75)
Five	/a/	48	43 (90)	48	48 (100)	48	43 (90)
(*n* = 423)	/i/	47	46 (98)	48	48 (100)	48	47 (98)
	/u, y/	47	41 (87)	46	46 (100)	43	43 (100)
	Overall	142	130 (92)	142	142 (100)	139	133 (96)
Adult	/a/	48	48 (100)	48	48 (100)	48	48 (100)
(*n* = 432)	/i/	48	48 (100)	48	48 (100)	48	48 (100)
	/u, y/	48	48 (100)	48	48 (100)	48	48 (100)
	Overall	144	144 (100)	144	144 (100)	144	144 (100)

Statistical analyses were performed in order to investigate whether there were significant differences in the accuracy rate between different age groups and places. Accuracy rates were first transformed into arcsine value to alleviate the ceiling and floor effects, and then a linear mixed-effects model was fitted. The dependent variable was the transformed accuracy rate, and the fixed factors were age (four levels: three-year-olds, four-year-olds, five-year-olds, and adults) and place of articulation (three levels: /ts^h^/, /tɕ^h^/, and /tȿ^h^/), and by-subject random slopes of place were also included in the model. The results are summarized in Supplementary Appendix C.

**FIGURE 2:**
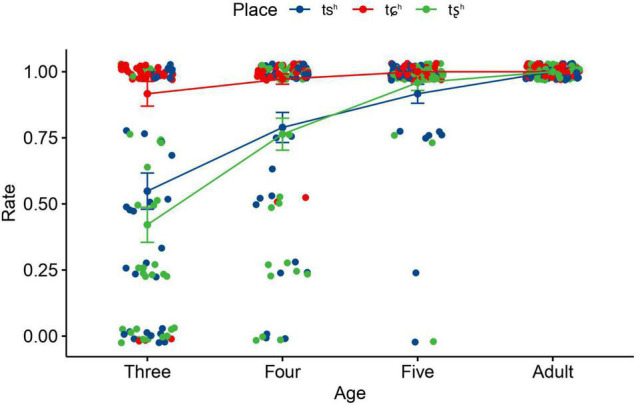
Means and standard errors of the accuracy rate of aspirated affricates as a function of age and place. Error bars: ± 1 standard error.

The results of linear mixed-effects model revealed that the main effects of age and place were statistically significant. In addition, significant two-way interaction effects were found between age and place, indicating that the effects of place varied in different age groups. Figure 2 demonstrates the interaction effect between age and place. It shows that the accuracy rate of each affricate improves with age. The accuracy rate of /tɕ^h^/ was much higher than that of /ts^h^/ and /tȿ^h^/ in the three- and four- year-olds. No statistically significant differences were found between /ts^h^/ and /tȿ^h^/ in each age group. In addition, no significant differences were found between child groups and adults for /tɕ^h^/, and it was until the age of five that children’s accuracy rate of /ts^h^/ and /tȿ^h^/ reached adult-like level. This suggested that /tɕ^h^/ was acquired at age three, prior to /ts^h^/ and /tȿ^h^/, both of which caused much more confusion in the three- and four-year-olds.

Table 3 presents the number and percentage of aspirated affricates judged to be incorrect by the transcriber. It can be seen that errors of /ts^h^/ and /tȿ^h^/ account for most of the total errors. As for the error types, children mostly substituted one affricate with another, and occasionally they used a fricative or a stop to replace the target affricate. In addition, the most frequent confusion was between /ts^h^/ and /tȿ^h^/, and only a rather small number of /ts^h^/ and /tȿ^h^/ were replaced by /tɕ^h^/. The production of both /ts^h^/ and /tȿ*^h^*/ involves the tongue tip, whereas the production of /tɕ^h^/ involves the tongue body, leading to the compatibility between /ts^h^/ and /tȿ^h^/, and incompatibility between /tɕ^h^/ and /ts^h^, tȿ^h^/.

**TABLE 3:** Number, percentage, and error types of aspirated affricates judged to be incorrect by the transcriber as a function of place.

Affricates	Affricate		Fricative		Stop	
	Error	Number (percentage)	Error	Number (proportion)	Error	Number (proportion)
/ts^h^/(*n* = 103)	/tȿ^h^/	92 (89)	/s/	1 (0.9)	/t^h^/	3 (2.9)
	/tɕ^h^/	5 (4.9)	/ȿ/	2 (1.9)		
/tɕ^h^/(*n* = 16)	/ts^h^/	7 (44)	/ɕ/	1 (6.3)	/t^h^/	1 (6.3)
	/tȿ^h^/	7 (44)				
/tȿ^h^/(*n* = 124)	/ts^h^/	96 (77)	/ȿ/	6 (4.8)	/t^h^/	7 (5.6)
	/tɕ^h^/	12 (9.7)	/s/	1 (0.8)		
	/tɕ/	1 (0.8)	/x/	1 (0.8)		

#### Children’s Unaspirated Affricates

Table 4 displays the number and percentage of unaspirated affricates judged to be correct by the transcriber as a function of age, place of articulation, and vowel context. In order to examine whether there were significant differences in the accuracy rate between different age groups and places, a similar linear mixed-effects model was built for the transformed accuracy rate with two fixed factors: age (four levels: three-year-olds, four-year-olds, five-year-olds and adults) and place of articulation (three levels: /ts^h^/, /tɕ^h^/, and /tȿ^h^/), and by-subject random slopes of place were also entered into the model. The results are summarized in Supplementary Appendix C.

**TABLE 4:** Number and percentage of unaspirated affricates judged to be correct by the transcriber as a function of age, place, and vowel context.

		/ts/		/tɕ/		/tȿ/	
Age	Vowel	Total	Number (percentage) Correct	Total	Number (percentage) Correct	Total	Number (percentage) Correct
Three	/a/	47	29 (62)	47	46 (98)	48	22 (46)
(*n* = 428)	/i/	48	34 (71)	48	41 (85)	48	11 (23)
	/u, y/	48	30 (63)	48	48 (100)	46	26 (57)
	Overall	143	93 (65)	143	135 (94)	142	59 (42)
Four	/a/	48	32 (67)	48	48 (100)	48	35 (73)
(*n* = 429)	/i/	48	48 (100)	48	48 (100)	47	32 (68)
	/u, y/	48	31 (65)	47	47 (100)	47	36 (77)
	Overall	144	111 (77)	143	143 (100)	142	103 (73)
Five	/a/	48	44 (92)	47	47 (100)	48	47 (98)
(*n* = 427)	/i/	46	46 (100)	47	47 (100)	47	47 (100)
	/u, y/	48	41 (85)	48	48 (100)	48	47 (98)
	Overall	142	131 (92)	142	142 (100)	143	141 (99)
Adult	/a/	48	48 (100)	48	48 (100)	48	48 (100)
(*n* = 432)	/i/	48	48 (100)	48	48 (100)	48	48 (100)
	/u, y/	48	48 (100)	48	48 (100)	48	48 (100)
	Overall	144	144 (100)	144	144 (100)	144	144 (100)

**FIGURE 3:**
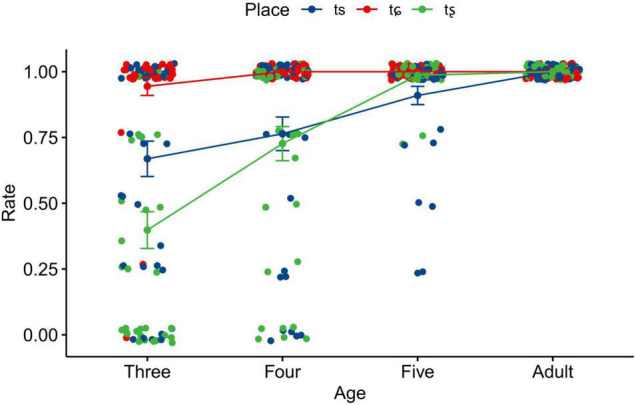
Means and standard errors of the accuracy rate of unaspirated affricates as a function of age and place. Error bars: ± 1 standard error.

The results of linear mixed-effects model revealed that the main effects of age and place were statistically significant. In addition, significant two-way interaction effects were found between age and place, indicating that the effects of place showed inconsistent patterns in different age groups. Figure 3 clearly illustrates the interaction effects between age and place. It can be seen that the accuracy rate of each affricate improves as age increases. The accuracy rate of /tɕ/ was much higher than that of /ts/ and /tȿ/ in the three- and four-year-olds. Statistically significant differences in the accuracy rate were also found between adults and two younger groups for both /ts/ and /tȿ/. It indicated that the three-year-olds had acquired /tɕ/, and it was until five years old that children reached adult-like levels for /ts/ and /tȿ/.

Table 5 describes the number and percentage of sounds judged to be incorrect for each affricate. It shows that the errors of /ts/ and /tȿ/ occupy a large portion of the total errors. The error types were mainly substitutions including affricates, fricatives, and stops, and the majority of errors were substitutions of another affricate with a different place of articulation. A small number of error types were of different manners such as fricatives and stops. It was worthwhile to note that 71% of /ts/ were substituted with /tȿ/ and 88% of /tȿ/ were substituted with /ts/. They seldom mispronounced /ts/ and /tȿ/ as /tɕ/, consistent with the pattern found for aspirated affricates, further indicating that the muscles used to produce /ts/ and /tȿ/ were not compatible with those used to produce /tɕ/.

**TABLE 5:** Number, percentage, and error types of unaspirated affricates judged to be incorrect by the transcriber as a function of place.

Affricate	Affricate		Fricative		Stop	
	Error	Number (percentage)	Error	Number (percentage)	Error	Number (percentage)
/ts/(*n* = 94)	/tȿ/	67 (71)	/s/	1 (1.1)	/t/	13 (13.8)
	/tɕ/	8 (8.5)				
	/ts^h^/	5 (5.3)				
/tɕ/(*n* = 8)	/tɕ^h^/	4 (50)				
	/ts/	3 (38)				
	/ts^h^/	1 (12)				
/tȿ/(*n* = 124)	/ts/	109 (88)			/t/	8 (6.5)
	/tɕ/	4 (3.2)				
	/tȿ^h^/	3 (2.4)				

### Acoustic Analysis

#### Children’s Aspirated Affricates

To examine when children could differentiate three aspirated affricates in terms of place of articulation, two linear mixed-effects models were fitted on F2 onset and spectral mean, respectively. Two fixed factors are age (three levels: the three-year-olds, the four-year-olds, and the five-year-olds) and place of articulation (three levels: /ts^h^/, /tɕ^h^/, and /tȿ^h^/), and by-subject random slopes for the effect of place were included as well. The results are summarized in Supplementary Appendix D.

**FIGURE 4:**
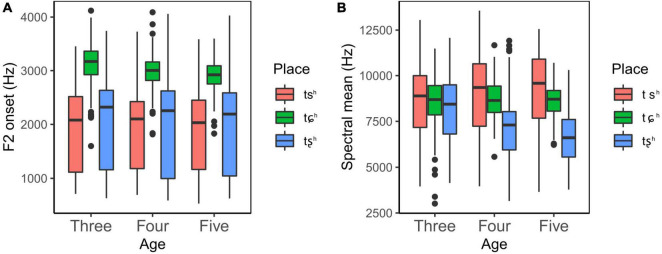
Box plots of F2 onset and spectral mean for aspirated affricates produced by children as a function of place and age. Each box describes the lower quartile, median, and upper quartile.

Figure 4A shows F2 onset for three aspirated affricates across age groups. The linear mixed-effects model yielded a significant main effect of place, but no significant main effect of age was observed. In addition, no significant interaction effect was found between age and place, indicative of consistent pattern in each age group. *Post hoc* analyses revealed that there were significant differences between /tɕ^h^/ and the other two aspirated affricates in each age group, suggesting that children began to differentiate /tɕ^h^/ from the other two aspirated affricates at the age of three in the dimension of F2 onset, as shown in Figure 4A.

The obtained values of spectral mean are shown in Figure 4B. The results of the linear mixed-effects model showed a significant main effect of place. However, no significant main effect of age was found. In addition, significant interaction effects were found between age and place. These interactions indicated that the effect of place was not consistent in different age groups. Given that this study was designed to examine when children could differentiate affricates in terms of place of articulation, further simple effects analysis was performed to investigate the interaction effects between age and place. It showed that there were no statistically significant differences between aspirated affricates in terms of place of articulation in the three-year-olds. In the four-year-olds, there were statistically significant differences between /tɕ^h^/ and the other two aspirated affricates, whereas no significant differences were found between /ts^h^/ and the other two posterior ones, indicating that children could separate /tȿ^h^/ from /ts^h^/ and /tɕ^h^/ at this stage, as illustrated in Figure 4B. These results implied that /tȿ^h^/ was acquired at the age of four in the dimension of spectral mean.

#### Children’s Unaspirated Affricates

Similarly, two separate linear mixed-effects models were constructed using two parameters (F2 onset and spectral mean). In these models, the fixed factors are age (three levels: the three-year-olds, the four-year-olds, and the five-year-olds) and place of articulation (three levels: /ts/, /tɕ/, and /tȿ/). In addition, by-subject random slopes for the effect of place were entered into the models. The results are summarized in Supplementary Appendix E.

**FIGURE 5:**
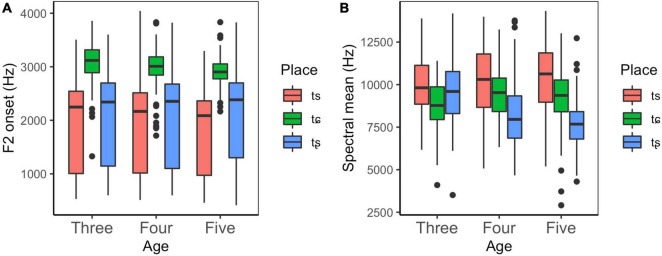
Box plots of F2 onset and spectral mean for unaspirated affricates produced by children as a function of place and age. Each box describes the lower quartile, median, and upper quartile.

As displayed in Figure 5A, F2 onset of /tɕ/ is much higher than that of /ts/ and /tȿ/ across different age groups. The results of linear mixed-effects model revealed that there existed a significant main effect of place, but no significant main effect was found for age. Moreover, no significant interaction effects were found between age and place. Subsequent *post hoc* analysis showed that there were significant differences between /tɕ/ and the other two in each age group. These results suggested that children aged three began to make a distinction between /tɕ/ and /ts, t/ in the dimension of F2 onset.

With respect to spectral mean, Figure 5B shows that spectral mean declines as the constriction point moves from the anterior part to the posterior part, with /ts/ having the highest spectral mean, followed by /tɕ/, then /tȿ/. The results of a linear mixed-effects model revealed that the main effect of place was found to be statistically significant, whereas no significant main effect of age was reported. It was also found that there were significant interaction effects between age and place, indicating that the effect of place showed different patterns in each age group. Further simple effects analysis showed that there were statistically significant differences between /tȿ/ and /ts, tɕ/ in the four-year-olds, and between all places in the five-year-olds, indicating that children aged four began to distinguish /tȿ/ from /ts/ and /tɕ/ and could distinguish all three unaspirated affricates at age five.

## Discussion

This study sought to investigate the phonological development of Mandarin voiceless affricates based on transcription and acoustic methods. Both methods revealed that alveopalatal affricates were acquired earlier than retroflex and alveolar, regardless of aspiration. However, the two measures diverged from each other in terms of the acquisition order of retroflex and alveolar affricates. The former revealed that both retroflex and alveolar affricates were acquired at age five, regardless of aspiration. However, the latter differed in that retroflex was acquired earlier than alveolar affricates. In addition, the error analysis revealed evident language-specific patterns.

Our findings are consistent with an array of previous studies (So and Dodd, 1995; Hua and Dodd, 2000; Si, 2006; Kim and Stoel-Gammon, 2011). Hua and Dodd (2000) reported earlier acquisition of alveopalatal affricates and later acquisition of alveolar affricates, diverging from the universal tendencies. Jakobson’s laws of implication postulated that place of articulation plays a prominent role in phonological development, namely, front consonants are acquired earlier compared with back consonants, and sounds frequently observed in world’s languages are acquired earlier than sounds that occur less commonly. The observed earlier acquisition of alveopalatal affricates forms sharp contrast to Jakobson’s assumption, indicating that the phonological acquisition order could not be exclusively determined in terms of place of articulation and the phoneme distribution in world’s languages (Jakobson, 1968; Locke, 1983) because of the relative backness and the rarity of alveopalatal affricates in world’s languages compared with the other two revealed by UCLA Phonological Segment Inventory Database (Maddieson, 1984; Ladefoged and Maddieson, 1996). Moreover, the language-specific phoneme frequencies also failed to offer a satisfactory explanation because the frequency of aspirated alveopalatal affricates (13,391) is slightly higher than that of aspirated retroflex (13,156) based on the calculation of phoneme frequencies in the phonetic transcriptions of the Lancaster Corpus of Mandarin Chinese (McEnery and Xiao, 2004). Instead, the earlier acquisition of alveopalatal affricates could be readily accounted for by the oromotor maturation hypothesis, which states that it is relatively easier for children to control the muscles related to the elevation of the tongue body than those used to raise the tongue tip, leading to the earlier acquisition of alveopalatal affricates compared with retroflex and alveolar affricates. Similar developmental patterns were also found in the acquisition of Mandarin sibilant fricatives (Li and Munson, 2016). However, it can be pointed out that that the tongue differed greatly from other body parts because of its highly sophisticated muscular hydrostat without any skeletal structures (Kier and Smith, 1985). Therefore, the relationship between tongue maturation and the developmental sequence of control might be much more complex than what the proximal-distal principle describes (Li and Munson, 2016).

It is particularly worthwhile to note that the transcription and acoustic methods differed in the acquisition order of retroflex and alveolar affricates. The difference is presumably due to the inadequate descriptive power of the transcription analysis based on adult’s categorical perception (Liberman et al., 1957). Transcribers might overlook the subtle differences between two sounds which correspond to a single category in adults’ mental lexicon (Li et al., 2009). Human perception is based on the original speech sounds, while the acoustic comparisons are based on specific/restricted acoustic parameters. More importantly, the acoustic analysis is performed on the group difference, while perceptual transcription is made on individual tokens. In addition, it is also likely that the observed simultaneous acquisition of retroflex and alveolar affricates based on the transcription analysis is an artifact of the wide age intervals in children, and the relatively insufficient number of tokens might also have obscured the actual phonological development of affricates. Therefore, a more systematic study with a narrower age interval may offer us an in-depth understanding of the speech sound acquisition sequence.

Furthermore, the acoustic analysis offered more detailed developmental trajectories of affricates. The earlier acquisition of retroflex affricates than alveolar affricates revealed by the acoustic analysis could be accounted for by the phoneme frequency in the ambient language. Retroflex occurs more frequently compared with alveolar affricates. To be specific, the frequency of aspirated retroflex affricates (13,156 for /tȿ^h^/ and 28,244 for /tȿ/) is about twice that of aspirated alveolar affricates (6,009 for /ts^h^/ and 15,168 for /ts/) on the basis of the phonetic transcriptions of the Lancaster Corpus of Mandarin Chinese (McEnery and Xiao, 2004). The frequently occurring sounds might play a more important role in the early stage of phonological development in younger children. Accordingly, children should have more opportunities to hear these sounds as different phonemes in a given language (Ingram, 1988b; Ma et al., 2021). This will lead to repeated activation of a simulated neural network, which, in turn, facilitates learning (Plaut and Kello, 1999). Similar findings were also found in previous studies of Mandarin sibilant fricatives. Li and Munson (2016) investigated the acquisition order of Mandarin sibilant fricatives, suggesting that retroflex fricatives were acquired before alveolar fricatives in Mandarin-speaking children aged 2–5 years old. This acquisition order was interpreted by language-specific phoneme frequencies as well. Taken together, an integrated theoretical model that incorporates oromotor development and language-specific phoneme frequencies could predict the observed sequence in the phonological development of Mandarin affricates.

In addition, the robustness of F2 onset to separate alveopalatal affricates from retroflex and alveolar affricates provides supporting evidence for previous perceptual studies (Nittrouer, 1992, 2002; Nittrouer and Miller, 1997; Mayo and Turk, 2004). They found that in the identification of the /s-vowel/-/∫-vowel/ contrast based on formant transition cues and the frequency of frication noise, younger children attend more to transition cues than adults, and similar patterns are reported in many other studies of the perception of the /s-vowel/-/∫-vowel/ contrast, whereas adults and old children rely more on the steady fricative-internal cues (Nittrouer, 1992; Nittrouer and Miller, 1997; Watson, 1997; Mayo et al., 2003). However, the present study is about speech production, not perception. We therefore could not claim that Mandarin-speaking children showed similar perceptual bias toward transition cues like English-speaking children. Perceptual studies are warranted to examine whether such perceptual bias also extends to Mandarin-speaking children.

Finally, the error pattern also exhibited language-specific patterns. Prior studies revealed that children frequently substitute stops for affricates, particularly in children of a younger age due to the articulatory complexity of affricates (Locke, 1983; Kim and Stoel-Gammon, 2011). Our findings demonstrated that substitution of stops for affricates occurs less frequently, and affricates are frequently substituted by other affricates. It is likely that children have learned certain speech skills to produce affricates, although their articulatory capacity is not mature. The decreasing number of errors across age groups suggests that they are continuously fine-tuning their motor speech abilities while undergoing anatomical and physiological development toward maturity (Yu et al., 2015; Ma et al., 2018). In addition, within a specific sound class, previous research showed that sounds produced in the back of the oral cavity are often replaced by those produced in the anterior part of the oral cavity, and the front sounds are relatively easier to be learned compared with back sounds. For example, the anterior sibilant fricative /s/ is universally easier than its post-alveolar counterpart /∫/ (Locke, 1983). In the current study, the error pattern revealed that children frequently substituted aspirated alveolar affricates for retroflex or vice versa, though the acoustic analysis revealed earlier acquisition of aspirated retroflex compared with alveolar ones. They seldom substituted alveopalatal affricates for retroflex and alveolar affricates, indicating that the muscles used for alveopalatals were not compatible with those for retroflex and alveolar affricates (Li and Munson, 2016). Furthermore, our findings provide evidence for the role of oromotor maturation in the phonological development in Mandarin-speaking children.

Our findings therefore suggest that phonological development in children is constrained by a combination of factors, such as oromotor maturation and language-specific phoneme frequencies. Oromotor maturation could readily account for the earlier acquisition of alveopalatal affricates, and language-specific phoneme frequencies could explain the earlier acquisition of retroflex than alveolar affricates. However, it is not easy to determine the relative contribution of each factor to phonological development. This study establishes current research as a productive direction for further examination of child speech development. However, there are several limitations and further research can be undertaken following these directions. First, in addition to the current cross-sectional investigation, longitudinal studies are warranted to further our understanding of speech development of individuals. Second, it is of interest to examine whether the same speech developmental patterns will be found in spontaneous speech production of isolated words or conversational speech. Third, the target structure can be extended to other phoneme types such as nasals and approximants, to have a panoramic view of child speech development. Fourth, the findings should be evaluated by large-scale studies in the future including more children aged less than 3 years old with a smaller age interval, which could enable researchers to scrutinize more specific phonological acquisition order in children.

## Conclusion

In a nutshell, the transcription and acoustic analyses of the study, while explorative in nature, shed light on the phonological acquisition order and provide a solid foundation for more systematic investigations of the phonological development in Mandarin-speaking children. These findings suggest that child speech development is a complex and dynamic process, constrained by both biological and environmental factors. No single factor proposed by current theoretical frameworks could provide a uniform account for the phonological development of Mandarin voiceless obstruents. Therefore, an integrated model incorporating various factors is needed to account for the observed sound acquisition order, though the relative weighting of each factor differs.

## Data Availability Statement

The raw data supporting the conclusions of this article will be made available by the authors, without undue reservation.

## Ethics Statement

The studies involving human participants were reviewed and approved by Ethics Committee of Hunan University. Written informed consent to participate in this study was provided by the participants’ legal guardian/next of kin.

## Author Contributions

JM formulated the research questions, conducted statistical analysis, and wrote the draft of the manuscript. JZ designed the experimental procedure. YW collected data. XC supervised the whole process. All authors have approved the final version of the manuscript.

## Conflict of Interest

The authors declare that the research was conducted in the absence of any commercial or financial relationships that could be construed as a potential conflict of interest.

## Publisher’s Note

All claims expressed in this article are solely those of the authors and do not necessarily represent those of their affiliated organizations, or those of the publisher, the editors and the reviewers. Any product that may be evaluated in this article, or claim that may be made by its manufacturer, is not guaranteed or endorsed by the publisher.
