# Supplementary material for: The Phonological Development of Mandarin Voiceless Affricates in Three- to Five-Year-Old Children
Source: Front Psychol. 2022 Mar 10;13:809722. doi: 10.3389/fpsyg.2022.809722 (PMC8961029; doi:10.3389/fpsyg.2022.809722)
Supplement: Supplementary file 1 [file Table_1.docx]

Table A. Word list for the affricate production experiment.

| Affricate | Pinyin | IPA | English gloss |
| --- | --- | --- | --- |
| /ts^h^/ | cangying | /ts^h^ɑŋ.yiŋ/ | fly |
|  | ciwei | /ts^h^ɿ.wuei/ | hedgehog |
|  | cuping | /ts^h^u.p^h^iŋ/ | vinegar bottle |
| /ts/ | zangshui | /tsɑŋ.ʂuei/ | dirty water |
|  | zidan | /tsɿ.tan/ | bullet |
|  | zuqiu | /tsu.tɕ^h^iou/ | football |
| /tɕ^h^/ | qianbi | /tɕ^h^ɑŋ.pi/ | pencil |
|  | qiqiu | /tɕ^h^i.tɕ^h^iou/ | balloon |
|  | qunzi | /tɕ^h^yn.tsɿ/ | skirt |
| /tɕ/ | jiazi | /tɕa.tsɿ/ | clip |
|  | jidan | /tɕi.tan/ | egg |
|  | juzi | /tɕy.tsɿ/ | orange |
| /tʂ^h^/ | chazi | /tʂ^h^a.tsɿ/ | fork |
|  | chifan | /tʂ^h^ʅ.fan/ | eating |
|  | chushi | /tʂ^h^u.ʂʅ/ | chef |
| /tʂ/ | zhadan | /tʂa.tan/ | bomb |
|  | zhibei | /tʂʅ.pei/ | paper cup |
|  | zhuzi | /tʂu.tsɿ/ | bamboo |
