# Supplementary material for: The Phonological Development of Mandarin Voiceless Affricates in Three- to Five-Year-Old Children
Source: Front Psychol. 2022 Mar 10;13:809722. doi: 10.3389/fpsyg.2022.809722 (PMC8961029; doi:10.3389/fpsyg.2022.809722)
Supplement: Supplementary file 2 [file Table_2.docx]

| Affricates | Parameter | Factor | *df1* | *df2* | *F* | *p* |
| --- | --- | --- | --- | --- | --- | --- |
| Aspirated Affricate | F2 onset | Place | 2 | 16 | 81.66 | *** |
|  | Spectral mean | Place | 2 | 11 | 40.03 | *** |
| Unaspirated Affricate | F2 onset | Place | 2 | 16 | 58.82 | *** |
|  | Spectral mean | Place | 2 | 11 | 131.05 | *** |

Table B.1. Results of linear mixed effects model with F2 onset and spectral mean of aspirated and unaspirated affricates in adults.

Note: R code: F2onset/Spectral mean∼Place +(1+Place|Subj), data). **p* < 0 .05. ***p* < 0.01. ****p* < 0.001.

Table B.2 Results of pairwise comparison on F2 onset and spectral mean of aspirated and unaspirated affricates for place contrasts in adults.

| Parameter | Place contrast | *β* | *SE* | *df* | *t* | *p* |
| --- | --- | --- | --- | --- | --- | --- |
| F2 onset | ts^h^-tɕ^h^ | -830 | 78 | 11 | -10.71 | *** |
|  | ts^h^-tʂ^h^ | -128 | 52 | 11 | -2.49 | 0.07 |
|  | tɕ^h^-tʂ^h^ | 702 | 56 | 11 | 12.637 | *** |
|  | ts-tɕ | -754 | 71 | 11 | -10.60 | *** |
|  | ts-tʂ | -257 | 47 | 11 | -5.51 | *** |
|  | tɕ-tʂ | 497 | 52 | 11 | 9.51 | *** |
| Spectral mean | ts^h^-tɕ^h^ | 1407 | 222 | 11 | 6.35 | *** |
|  | ts^h^-tʂ^h^ | 2669 | 308 | 11 | 8.66 | *** |
|  | tɕ^h^-tʂ^h^ | 1262 | 273 | 11 | 4.62 | ** |
|  | ts-tɕ | 1968 | 203 | 11 | 9.68 | *** |
|  | ts-tʂ | 3812 | 236 | 11 | 16.19 | *** |
|  | tɕ-tʂ | 1844 | 204 | 11 | 9.05 | *** |
