# Supplementary material for: The Phonological Development of Mandarin Voiceless Affricates in Three- to Five-Year-Old Children
Source: Front Psychol. 2022 Mar 10;13:809722. doi: 10.3389/fpsyg.2022.809722 (PMC8961029; doi:10.3389/fpsyg.2022.809722)
Supplement: Supplementary file 3 [file Table_3.docx]

Table C.1 Results of linear mixed effects model with the transformed accuracy rate as a function age and place for aspirated and unaspirated affricates.

| Affricates | Parameter | Factor | *df1* | *df2* | *F* | *p* |
| --- | --- | --- | --- | --- | --- | --- |
| Aspirated  affricates | Rate | Age | 3 | 46 | 28.92 | *** |
|  |  | Place | 2 | 46 | 18.45 | *** |
|  |  | Age × Place | 6 | 46 | 5.76 | *** |
| Unaspirated  affricates | Rate | Age | 3 | 54 | 35.36 | *** |
|  |  | Place | 2 | 49 | 25.57 | *** |
|  |  | Age × Place | 6 | 49 | 7.53 | *** |

Note: R code: (Rate~Age*Place+(1+Subject|Subj), data). **p* < 0 .05. ***p* < 0.01. ****p* < 0.001.

Table C.2 Results of pairwise comparison on the transformed accuracy rate of aspirated and unaspirated affricates for place contrasts in each age group.

| Age Group | Parameter | Place contrast | *β* | *SE* | *df* | *t* | *p* |
| --- | --- | --- | --- | --- | --- | --- | --- |
| Three | Rate | ts^h^-tɕ^h^ | -0.67 | 0.12 | 44 | -5.65 | *** |
|  |  | ts^h^-tʂ^h^ | 0.21 | 0.13 | 44 | 1.62 | 0.25 |
|  |  | tɕ^h^-tʂ^h^ | 0.88 | 0.12 | 44 | 1.11 | *** |
| Four |  | ts^h^-tɕ^h^ | -0.33 | 0.12 | 44 | -2.81 | * |
|  |  | ts^h^-tʂ^h^ | 0.03 | 0.13 | 44 | 0.23 | 0.97 |
|  |  | tɕ^h^-tʂ^h^ | 0.36 | 0.12 | 44 | 2.94 | * |
| Five |  | ts^h^-tɕ^h^ | -0.18 | 0.12 | 44 | -1.52 | 0.29 |
|  |  | ts^h^-tʂ^h^ | -0.10 | 0.13 | 44 | -0.77 | 0.73 |
|  |  | tɕ^h^-tʂ^h^ | 0.08 | 0.12 | 44 | 0.68 | 0.78 |
| Adult |  | ts^h^-tɕ^h^ | 0 | 0.12 | 44 | 0 | 1 |
|  |  | ts^h^-tʂ^h^ | 0 | 0.13 | 44 | 0 | 1 |
|  |  | tɕ^h^-tʂ^h^ | 0 | 0.12 | 44 | 0 | 1 |
| Three | Rate | ts-tɕ | -0.49 | 0.12 | 44 | -4.19 | *** |
|  |  | ts-tʂ | 0.44 | 0.16 | 44 | 2.83 | * |
|  |  | tɕ-tʂ | 0.93 | 0.12 | 44 | 7.65 | *** |
| Four |  | ts-tɕ | -0.42 | 0.12 | 44 | -3.57 | ** |
|  |  | ts-tʂ | 0.08 | 0.16 | 44 | 0.51 | 0.87 |
|  |  | tɕ-tʂ | 0.50 | 0.12 | 44 | 4.09 | *** |
| Five |  | ts-tɕ | -0.19 | 0.12 | 44 | -1.63 | 0.24 |
|  |  | ts-tʂ | -0.15 | 0.16 | 44 | -0.97 | 0.60 |
|  |  | tɕ-tʂ | 0.04 | 0.12 | 44 | 0.33 | 0.94 |
| Adult |  | ts-tɕ | 0 | 0.12 | 44 | 0 | 1 |
|  |  | ts-tʂ | 0 | 0.16 | 44 | 0 | 1 |
|  |  | tɕ-tʂ | 0 | 0.12 | 44 | 0 | 1 |

Note: **p* < 0 .05. ***p* < 0.01. ****p* < 0.001.

Table C.3 Results of pairwise comparison on the transformed accuracy rate of aspirated and unaspirated affricates between age groups for each affricate.

| Place | Parameter | Group contrast | *β* | *SE* | *df* | *t* | *p* |
| --- | --- | --- | --- | --- | --- | --- | --- |
| ts^h^ | Rate | Three-Four | -0.41 | 0.14 | 44 | -2.93 | * |
|  |  | Three-Five | -0.62 | 0.14 | 44 | -4.44 | *** |
|  |  | Three-Adult | -0.80 | 0.14 | 44 | -5.72 | *** |
|  |  | Four-Five | -0.21 | 0.14 | 44 | -1.51 | 0.44 |
|  |  | Four-Adult | -0.39 | 0.14 | 44 | -2.79 | 0.04 |
|  |  | Five-Adult | -0.18 | 0.14 | 44 | -1.29 | 0.58 |
| tɕ^h^ |  | Three-Four | -0.07 | 0.08 | 44 | -0.89 | 0.81 |
|  |  | Three-Five | -0.13 | 0.08 | 44 | -1.59 | 0.40 |
|  |  | Three-Adult | -0.13 | 0.08 | 44 | -1.59 | 0.40 |
|  |  | Four-Five | -0.06 | 0.08 | 44 | -0.71 | 0.90 |
|  |  | Four-Adult | -0.06 | 0.08 | 44 | -0.71 | 0.90 |
|  |  | Five-Adult | 0 | 0.08 | 44 | 0 | 1 |
| tʂ^h^ |  | Three-Four | -0.59 | 0.14 | 44 | -4.06 | ** |
|  |  | Three-Five | -0.92 | 0.14 | 44 | -6.39 | *** |
|  |  | Three-Adult | -1.01 | 0.14 | 44 | -6.97 | *** |
|  |  | Four-Five | -0.34 | 0.14 | 44 | -2.33 | 0.11 |
|  |  | Four-Adult | -0.42 | 0.14 | 44 | -2.91 | 0.03 |
|  |  | Five-Adult | -0.08 | 0.14 | 44 | -0.58 | 0.94 |
| ts | Rate | Three-Four | -0.17 | 0.14 | 44 | -1.23 | 0.61 |
|  |  | Three-Five | -0.40 | 0.14 | 44 | -2.83 | 0.03 |
|  |  | Three-Adult | -0.59 | 0.14 | 44 | -4.20 | *** |
|  |  | Four-Five | -0.23 | 0.14 | 44 | -1.61 | 0.39 |
|  |  | Four-Adult | -0.42 | 0.14 | 44 | -2.97 | * |
|  |  | Five-Adult | -0.19 | 0.14 | 44 | -1.36 | 0.53 |
| tɕ |  | Three-Four | -0.10 | 0.08 | 44 | -1.30 | 0.57 |
|  |  | Three-Five | -0.10 | 0.08 | 44 | -1.30 | 0.57 |
|  |  | Three-Adult | -0.10 | 0.08 | 44 | -1.30 | 0.57 |
|  |  | Four-Five | 0 | 0.08 | 44 | 0 | 1 |
|  |  | Four-Adult | 0 | 0.08 | 44 | 0 | 1 |
|  |  | Five-Adult | 0 | 0.08 | 44 | 0 | 1 |
| tʂ |  | Three-Four | -0.53 | 0.14 | 44 | -3.75 | * |
|  |  | Three-Five | -0.99 | 0.14 | 44 | -6.97 | *** |
|  |  | Three-Adult | -1.03 | 0.14 | 44 | -7.23 | *** |
|  |  | Four-Five | -0.46 | 0.14 | 44 | -3.22 | * |
|  |  | Four-Adult | -0.50 | 0.14 | 44 | -3.50 | ** |
|  |  | Five-Adult | -0.04 | 0.14 | 44 | -0.28 | 0.99 |
