# Supplementary material for: The Phonological Development of Mandarin Voiceless Affricates in Three- to Five-Year-Old Children
Source: Front Psychol. 2022 Mar 10;13:809722. doi: 10.3389/fpsyg.2022.809722 (PMC8961029; doi:10.3389/fpsyg.2022.809722)
Supplement: Supplementary file 4 [file Table_4.docx]

Table D.1. Results of linear mixed effects model with F2 onset and spectral mean of aspirated affricates in children.

| Affricate | Parameter | Factor | *df1* | *df2* | *F* | *p* |
| --- | --- | --- | --- | --- | --- | --- |
| Aspirated affricates | F2 onset | Age | 2 | 31 | 0.87 | 0.43 |
|  |  | Place | 2 | 424 | 310.32 | *** |
|  |  | Age × Place | 4 | 424 | 1.03 | 0.39 |
|  | Spectral mean | Age | 2 | 33 | 1.30 | 0.29 |
|  |  | Place | 2 | 33 | 53.39 | *** |
|  |  | Age × Place | 4 | 33 | 7.79 | *** |

Note: R code: F2onset/Spectral mean∼Age*Place+(1+Place|Subj), data). **p* < 0 .05. ***p* < 0.01. ****p* < 0.001.

Table D.2 Results of pairwise comparison on F2 onset and spectral mean of aspirated affricates for place contrasts in each age group.

| Age Group | Parameter | Place contrast | *β* | *SE* | *df* | *t* | *p* |
| --- | --- | --- | --- | --- | --- | --- | --- |
| Three  Four  Five | F2 onset | ts^h^-tɕ^h^ | -1070 | 48 | 33 | -22.37 | *** |
|  |  | ts^h^-tʂ^h^ | -77 | 47 | 33 | -1.62 | 0.25 |
|  |  | tɕ^h^-tʂ^h^ | 993 | 48 | 33 | 20.81 | *** |
| Three | Spectral mean | ts^h^-tɕ^h^ | 5 | 300 | 33 | 0.02 | 1 |
|  |  | ts^h^-tʂ^h^ | 404 | 319 | 33 | 1.27 | 0.42 |
|  |  | tɕ^h^-tʂ^h^ | 399 | 278 | 33 | 1.43 | 0.34 |
| Four |  | ts^h^-tɕ^h^ | 336 | 299 | 33 | 1.13 | 0.51 |
|  |  | ts^h^-tʂ^h^ | 2024 | 317 | 33 | 6.39 | *** |
|  |  | tɕ^h^-tʂ^h^ | 1687 | 276 | 33 | 6.11 | *** |
| Five |  | ts^h^-tɕ^h^ | 586 | 298 | 33 | 1.96 | 0.14 |
|  |  | ts^h^-tʂ^h^ | 2646 | 317 | 33 | 8.35 | *** |
|  |  | tɕ^h^-tʂ^h^ | 2060 | 276 | 33 | 7.46 | *** |

Note: **p* < 0 .05. ***p* < 0.01. ****p* < 0.001.
