# Supplementary material for: The Phonological Development of Mandarin Voiceless Affricates in Three- to Five-Year-Old Children
Source: Front Psychol. 2022 Mar 10;13:809722. doi: 10.3389/fpsyg.2022.809722 (PMC8961029; doi:10.3389/fpsyg.2022.809722)
Supplement: Supplementary file 5 [file Table_5.docx]

Table E.1. Results of linear mixed effects model with F2 onset and spectral mean of unaspirated affricates in children.

| Affricate | Parameter | Factor | *df1* | *df2* | *F* | *p* |
| --- | --- | --- | --- | --- | --- | --- |
| Unaspirated affricates | F2 onset | Age | 2 | 29 | 1.26 | 0.30 |
|  |  | Place | 2 | 1035 | 307.21 | *** |
|  |  | Age × Place | 4 | 1055 | 1.16 | 0.33 |
|  | Spectral mean | Age | 2 | 33 | 0.75 | 0.48 |
|  |  | Place | 2 | 33 | 23.02 | *** |
|  |  | Age × Place | 4 | 33 | 7.60 | *** |

Note: R code: F2onset/Spectral mean∼Age*Place+(1+Place|Subj), data). **p* < 0 .05. ***p* < 0.01. ****p* < 0.001.

Table E.2 Results of pairwise comparison on F2 onset and spectral mean of unaspirated affricates for place contrasts in each age group.

| Age Group | Parameter | Place contrast | *β* | *SE* | *df* | *t* | *p* |
| --- | --- | --- | --- | --- | --- | --- | --- |
| Three  Four  Five | F2 onset | ts-tɕ | -1058 | 37 | 33 | -28.6 | *** |
|  |  | ts-tʂ | -47 | 25 | 33 | -1.84 | 0.17 |
|  |  | tɕ-tʂ | 1011 | 33 | 33 | 31.02 | *** |
| Three | Spectral mean | ts-tɕ | 1109 | 383 | 34 | 2.90 | * |
|  |  | ts-tʂ | 422 | 416 | 34 | 1.02 | 0.57 |
|  |  | tɕ-tʂ | -686 | 337 | 34 | -2.04 | 0.12 |
| Four |  | ts-tɕ | 473 | 380 | 33 | 1.25 | 0.44 |
|  |  | ts-tʂ | 1781 | 412 | 33 | 4.32 | *** |
|  |  | tɕ-tʂ | 1308 | 336 | 33 | 3.90 | ** |
| Five |  | ts-tɕ | 1031 | 380 | 33 | 2.71 | * |
|  |  | ts-tʂ | 2639 | 412 | 33 | 6.41 | *** |
|  |  | tɕ-tʂ | 1608 | 335 | 33 | 4.80 | *** |

Note: **p* < 0 .05. ***p* < 0.01. ****p* < 0.001.
